# The effect of DPP-4 inhibitors, GLP-1 receptor agonists and SGLT-2 inhibitors on cardiorenal outcomes: a network meta-analysis of 23 CVOTs

**DOI:** 10.1186/s12933-022-01474-z

**Published:** 2022-03-16

**Authors:** Dario Giugliano, Miriam Longo, Simona Signoriello, Maria Ida Maiorino, Bruno Solerte, Paolo Chiodini, Katherine Esposito

**Affiliations:** 1 Department of Advanced Medical and Surgical Sciences, PhD of Translational Medicine, University of Campania Luigi Vanvitelli, Naples, Italy; 2 Division of Endocrinology and Metabolic Diseases, Department of Advanced Medical and Surgical Sciences, University of Campania Luigi Vanvitelli, Naples, Italy; 3 Medical Statistics Unit, Department of Mental Health and Public Medicine, University of Campania Luigi Vanvitelli, Naples, Italy; 4 Dipartimento di Medicina Interna e Terapia Medica, Università Degli Studi Di Pavia, Pavia, Italy

**Keywords:** Cardiovascular outcome trials, DPP-4 inhibitors, GLP-1 receptor agonists, SGLT-2 inhibitors, Network meta-analysis

## Abstract

**Background:**

Glucagon-like peptide-1 receptor agonists (GLP-1RA) and sodium glucose co-transporter-2 (SGLT-2) inhibitors reduce cardiorenal outcomes. We performed a network meta-analysis to compare the effect on cardiorenal outcomes among GLP-1 RAs, SGLT-2 inhibitors and dipeptidyl peptidase-4 (DPP-4) inhibitors.

**Methods:**

We searched the PUBMED, Embase and Cochrane databases for relevant studies published up until 10 December 2021. Cardiovascular and renal outcome trials reporting outcomes on GLP-1RA, SGLT-2 inhibitors and DPP-4 inhibitors in patients with or without type 2 diabetes mellitus were included. The primary outcome was major adverse cardiovascular events (MACE); other outcomes were cardiovascular and total death, nonfatal myocardial infarction (MI), nonfatal stroke, hospitalization for heart failure (HHF), and renal outcome.

**Results:**

Twenty-three trials enrolling a total number of 181,143 participants were included. DPP-4 inhibitors did not lower the risk of any cardiorenal outcome when compared with placebo and were associated with higher risks of MACE, HHF, and renal outcome when compared with the other two drug classes. SGLT-2 inhibitors significantly reduced cardiovascular (RR = 0.88) and total (RR = 0.87) death, as compared with DPP-4 inhibitors, while GLP-1 RA reduced total death only (RR = 0.87). The comparison between GLP-1RA and SGLT-2 inhibitors showed no difference in their risks of MACE, nonfatal MI, nonfatal stroke, CV and total death; SGLT-2 inhibitors were superior to GLP-1RA in reducing the risk of HHF and the renal outcome (24% and 22% lower risk, respectively). Only GLP-1RA reduced the risk of nonfatal stroke (RR = 0.84), as compared with placebo. There was no head-to-head trial directly comparing these antidiabetic drug classes.

**Conclusions:**

SGLT-2 inhibitors and GLP-1RA are superior to DPP-4 inhibitors in reducing the risk of most cardiorenal outcomes; SGLT-2 inhibitors are superior to GLP-1RA in reducing the risk of HHF and renal events; GLP-1RA only reduced the risk of nonfatal stroke. Both SGLT-2 inhibitors and GLP-1RA should be the preferred treatment for type 2 diabetes and cardiorenal diseases.

**Supplementary Information:**

The online version contains supplementary material available at 10.1186/s12933-022-01474-z.

## Introduction

Diabetes has reached alarming levels. More than half a billion people are living with diabetes worldwide and it is estimated that by 2045 there will be 693 million people diagnosed with the condition [1]. In addition, 541 million people are estimated to have impaired glucose tolerance, which brings to more than one billion the number of people living with subtle or frank alteration of glucose metabolism. The most common cardiovascular (CV) manifestations in patients with type 2 diabetes are heart failure (HF), stable angina, nonfatal myocardial infarction (MI), peripheral arterial disease and ischemic stroke [2–4] which accounts for 60% of their deaths [5]. Chronic kidney disease (CKD) also contributes to the major disease burden of patients with type 2 diabetes [6]. Therefore, the esteem for diabetes-related causes of death in 2021 have been updated to 6.7 million [1].

The quite unexpected positive cardiorenal outcomes that have emerged from the cardiovascular outcomes trials (CVOTs) with both glucagon like peptide-1 receptors agonists (GLP-1RA) and sodium-glucose co-transporter 2 (SGLT-2) inhibitors have produced a shift of diabetes management from the meticulous glycemic control alone to the simultaneous improvement of CV outcomes. Therefore, almost all scientific guidelines now recommend specific treatments for patients with type 2 diabetes based on CVOTs data, namely adding to metformin a SGLT-2 inhibitor or a GLP-1RA with proven CV disease benefit in patients with type 2 diabetes and CV disease, established kidney disease, or HF [7–10]. Owing to their lack of efficacy on cardiorenal outcomes, dipeptidyl-peptidase (DPP-4) inhibitors are only recommended to control hyperglycemia. There are also other practical reasons to prescribe these drugs in people with type 2 diabetes, because all three classes are associated with relatively low risk of hypoglycemic events, while patients treated with GLP-1RA and SGLT-2 inhibitors may also benefit from weight loss [11].

The cardiorenal effects of both GLP-1RA and SGLT-2 inhibitors can be summarized as follows: GLP-1RA have moderate benefits on MACE (major cardiovascular events), may reduce all-cause mortality and hospitalization for HF (HHF), and have a robust effect in reducing the incidence of macroalbuminuria [12]; SGLT-2 inhibitors cause robust reduction of HHF and the renal outcome, and moderate reduction of CV or total deaths and MACE [13]. However, data directly comparing GLP-1RA and SGLT-2 inhibitors are lacking, which limit the choice of clinicians between them. An indirect help may come from network meta-analysis, which is a technique for comparing three or more interventions simultaneously in a single analysis by combining both direct and indirect evidence across a network of studies. Previous similar exercises [14, 15] have indicated that SGLT-2 inhibitors are superior to GLP-1RA in reducing HHF and the renal outcome. However, new data on this subject continue to be released which adds new evidence to the therapeutic scenario.

In this current network meta-analysis, we evaluated 23 CVOTs, including the most recently published trials AMPLITUDE-O and EMPEROR-P. We aimed to compare the relative efficacy of SGLT-2 inhibitors, GLP-1RA and DPP-4 inhibitors in terms of cardiorenal outcomes.

## Methods

### Search strategy and study selection

This meta-analysis was performed according to PRISMA (Preferred Reporting Items for Systematic Reviews and Meta-Analyses) guidelines [16]. The protocol has not been registered in any platform. We searched PubMed, EMBASE and the Cochrane Database of Systematic Reviews to identify English-language studies published up until 10 December 2021. The search was limited to outcome trials evaluating the efficacy of DPP-4 inhibitors, GLP-1RA and SGLT-2 inhibitors on cardiovascular, heart failure or renal outcomes in adult patients with or without type 2 diabetes. The terms used for the research were “DPP-4 inhibitors”, “GLP-1 receptor agonists”, “SGLT-2 inhibitors”, “MACE”, “heart failure”, “renal outcome”, “total death” “cardiovascular death” and their synonyms and related keywords. The search was filtered to include only randomized controlled trials (RCTs) or meta-analyses of human data. The prespecified selection criteria included: (1) RCTs reporting desired cardiovascular or renal outcomes; (2) RCTs completed before the FDA guidance of 2008 [17] and (3) follow-up duration of at least 6 months.

### Data extraction and quality assessment

Data were extracted by D.G. and M.L., with conflicts over study inclusion resolved by consensus. Results in trial reports (primary trial results and subsequent secondary publications), and their accompanying Additional file 1, were used as the primary source of information. The retrieved data included study characteristics, characteristics of patients, interventions, and outcome measures, that included the hazard ratios (HR) and confidence intervals (CI) for cardiorenal outcomes. The Cochrane Collaboration Risk-of-Bias tool was used for quality assessment of the RCTs [18], including sequence generation, allocation concealment, blinding, incomplete outcome data, and selective outcome reporting. Risk of bias was graded as unclear, high, or low.

### Outcomes

The primary outcome in our network meta-analysis was MACE, defined as the composite of cardiovascular mortality, nonfatal myocardial infarction (MI), and nonfatal stroke. Co-primary outcomes were cardiovascular and total death. Secondary outcomes included nonfatal MI, nonfatal stroke, HHF and renal composite outcome, defined in most trials as a composite of progression to end-stage renal disease or a sustained decrease of at least 40% in estimated glomerular filtration rate (eGFR). The definitions of outcomes were those used in each trial.

### Data synthesis and analysis

The comparison of outcomes among the different glucose-lowering drug classes was made using a frequentist approach for direct and indirect treatment comparisons for each endpoint [19]. For all pair wise comparisons, Risk ratios (RRs) and 95% Confidence Interval (CI) were used as the meta-analytic measure of association between treatment and the incidence of events. The overall heterogeneity of all the comparisons was assessed using the I^2^ statistic, in which values of > 25%, > 50% and > 75% correspond to low, moderate, and high heterogeneity, respectively. There was no need to evaluate the inconsistency between the direct (any class of drugs vs another class) and indirect (any class of drugs vs placebo) effects in the network meta-analysis, as there was no trial that compared any two classes in all the trials. Publication bias was assessed with the Egger test [20]. The trim-and-fill method [21] was used to estimate the effect of publication bias, if any. The relative ranking of the different treatment was estimated for each outcome using p-score [22]. The network analysis was performed using R (version 4.1.2, updated by 21 November 2021) netmeta package. Other statistical analyses were conducted, and forest plots created using Stata software (version 16.0, Stata Corp., College Station, TX).

## Results

### Search and risk of bias

Of the 54 articles assessed for eligibility, 23 RCTs (References 1–23 in the Additional file 1) fulfilled the inclusion criteria and were included in the meta-analysis (Additional file 1: Fig. S1). Their characteristics are summarized in Table 1. The participants were all adults (> 18 years old) patients and all trials were multinational and sponsored by industry. The trials have been published between 2013 and 2021, with four trials published in 2021. All trials were of parallel group, double-blind design, and their mean duration ranged from 0.75 (SOLOIST-WHF) to 5.4 years (REWIND). The populations studied ranged in size from 1222 (SOLOIST-WHF) to 17,160 (DECLARE) and were of similar age (range 61 to 71.9 years). Of these trials, four compared DPP-4 inhibitors against placebo (SAVOR-TIMI 53, EXAMINE, TECOS, CARMELINA), eight compared GLP-1RA against placebo (ELIXA, LEADER, SUSTAIN-6, EXSCEL, HARMONY, REWIND, PIONEER 6, AMPLITUTE-O) and eleven compared SGLT-2 inhibitors against placebo (EMPA-REG, CANVAS, DECLARE, CREDENCE, DAPA-HF, DAPA CKD, VERTIS-CV, EMPEROR-R, SCORED, SOLOIST-WHF, EMPEROR-P). The participants in the studies were 43,522 with DPP-4 inhibitors, 60,080 with GLP-1RA, and 77,541 with SGLT-2 inhibitors, for a total number of 181,143 participants. At the present, there is still no published or in progress trial directly comparing the cardiorenal outcomes of these three classes of drugs. The primary outcomes for the 23 trials are also given in Table 1. According to the Cochrane Collaboration’s tool for assessing risk of bias, all trials met the criteria for low risk of bias (Additional file 1: Table S1, Fig. S2).

**Table 1:** Summary of the 23 CVOTs included

Trial/year of publication	Study drug/mean follow up (years)	Participants (n)	Age mean/median age (years)	Male sex (n, %)	Participants with prior CV disease (%)	Primary outcome	Study funder
DPP-4 inhibitors							
SAVOR-TIMI53/2013	Saxagliptin/2.1	16,492	65	11,050 (67%)	78	MACE	AstraZeneca
EXAMINE/2013	Alogliptin/1.5	5380	61	3658 (68%)	100	MACE	Takeda
TECOS/2015	Sitagliptin/3.0	14,523	65	10,311 (71%)	100	MACE	Merck
CARMELINA/2019	Linagliptin/2.2	6979	66	4788 (68.6%)	57	MACE	Boehringer/Lilly
GLP-1RA							
ELIXA, 2015	Lixisenatide/2.1	6068	60.3	3174 (69.3%)	100	MACE	Sanofi
LEADER/2016	Liraglutide/3.8	9340	64.3	6003 (64.3%)	81	MACE	NovoNordisk
SUSTAIN-6/2016	Semaglutide/2.1	3297	64.6	2002 (60.7%)	83	MACE	NovoNordisk
EXSCEL/2017	Exenatide/3.2	14,752	62	9149 (62%)	73.1	MACE	Amylin
HARMONY/2018	Albiglutide/1.6	9463	64.1	6569 (69.4%)	100	MACE	GlaxoSmithKline
REWIND/2019	Dulaglutide/5.4	9901	66.2	5312 (53.7%)	31.4	MACE	Boehringer/Lilly
PIONEER 6 /2019	Semaglutide/1.3	3183	66.0	2176 (68.4%)	84.7	MACE	NovoNordisk
AMPLITUDE-O/2021	Epfeglenatide/1.8	4076	64.5	2732 (67%)	89.6	MACE	SANOFI
SGLT-2 inhibitors							
EMPA-REG/2015	Empagliflozin/3.1	7020	63.2	5016 (71.5%)	99	MACE	Boehringer/Lilly
CANVAS/2017	Canagliflozin/2.4	10,142	63.2	6509 (64.2%)	66	MACE	Janssen
DECLARE/2019	Dapagliflozin/4.2	17,16	63.8	10,738 (62.6%)	40.6	MACE	AstraZeneca
CREDENCE/2019	Canagliflozin/2.6	4401	63	2907 (66.1%)	50.4	Composite renal	Janssen
DAPA-HF/2019	Dapagliflozin/1.5	4744	66	3131 (66%)	100	HHF or CV death	AstraZeneca
VERTIS-CV/2020	Ertugliflozin/3.5	8246	64.4	5769 (70%)	100	MACE	Merck
DAPA-CKD/2020	Dapagliflozin/2.4	4304	61.8	2879 (66.9%)	38	Composite renal	AstraZeneca
EMPEROR-R/2020	Empagliflozin/1.3	3730	66.8	2837 (76%)	100	HHF or CV death	Boehringer/Lilly
SCORED/2021	Sotagliflozin/1.3	10,584	69	5896 (55.7%)	NA	HHF or CV death	Sanofi/Lexicon
SOLOIST-WHF/2021	Sotagliflozin/0.75	1222	70	810 (63.3%)	100	HHF or CV death	Sanofi/Lexicon
EMPEROR-P/2021	Empagliflozin/2.2	5988	71.9	3317 (55.4%)	100	HHF or CV death	Boehringer/Lilly

*HHF* hospitalization for heart failure

### Outcomes

**Fig. 1:**
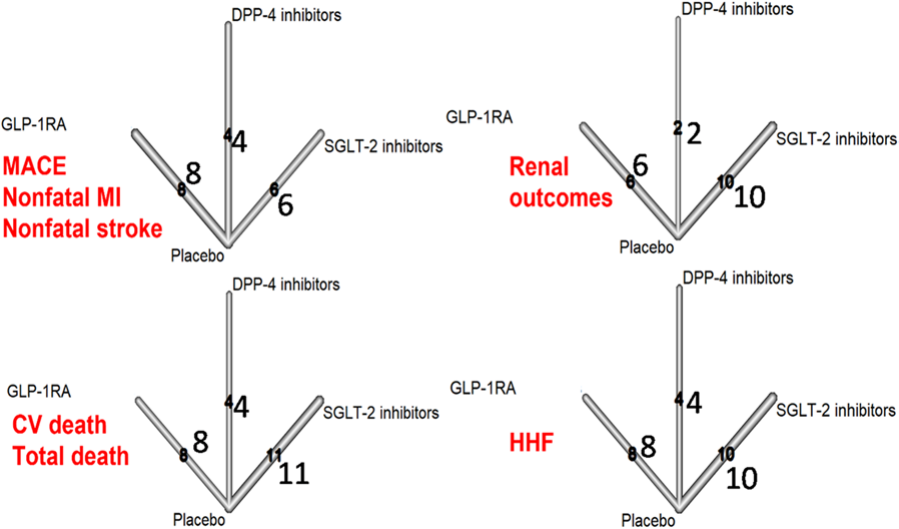
The network of comparisons for the outcomes. The numbers on the arrows indicate the number of comparisons with placebo for each outcome

**Fig. 2:**
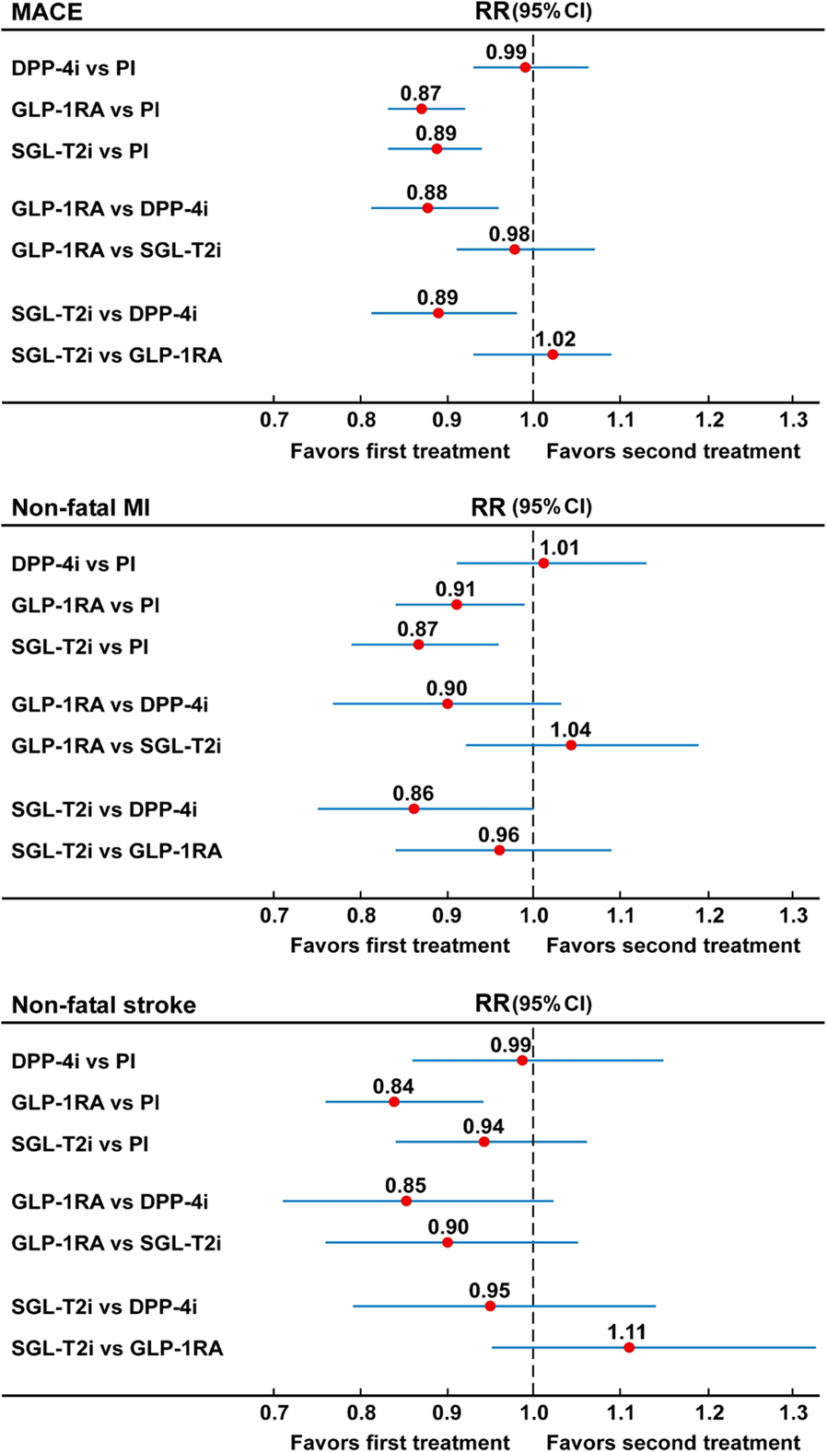
Forest plot of the network meta-analysis of MACE, nonfatal myocardial infarction, and nonfatal stroke. DPP-4i: dipeptydil-peptidase-4 inhibitors; Pl: placebo; GLP-1RA: glucagon-like peptide-1 receptor agonists; SGLT-2i: sodium-glucose cotransporter-2 inhibitors

Figure 1 shows the network of comparisons for the outcomes. Figure 2 and Additional file 1: Fig. S3 show the results for the outcome MACE: both GLP-1RA and SGLT-2 inhibitors were associated with a lower risk for MACE (13% and 11%, respectively) as compared with placebo, and a lower risk for MACE as compared with DPP-4 inhibitors (12% and 11%, respectively). Moreover, the comparison between DPP-4 inhibitors and placebo demonstrated no difference in their risk for MACE (RR = 0.99, 95% CI 0.93–1.06). The comparison between GLP-1RA and SGLT-2 inhibitors also show no difference in their risk for MACE (RR = 0.98, 95% CI 0.91–1.07). P-rank scores show that GLP-1RA were ranked first in reducing the risk of MACE (88.8%) while SGLT-2 inhibitors were ranked second (77.5%) (Table S2). The overall heterogeneity for MACE was low (I^2^ = 15.3%, 95% uncertainty intervals, 0 and 52.3%) (Table S3). There was some evidence of asymmetry for MACE (Egger test, P = 0.059), suggesting a potential threat of publication bias; however, the trim-and-fill method indicated that this publication bias did not change the statistical significance of the estimate.

**Fig. 3:**
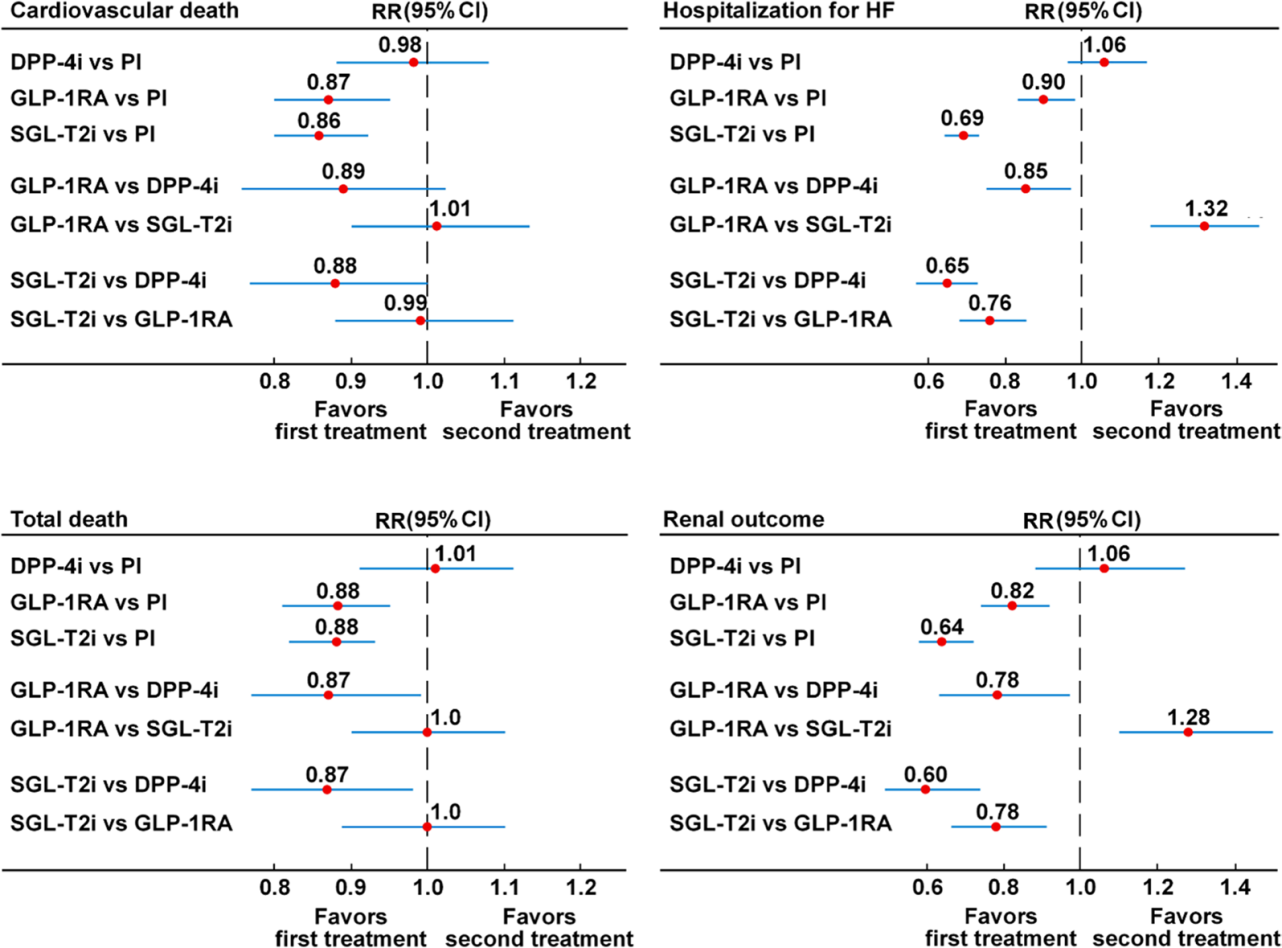
Forest plot of the network meta-analysis of cardiovascular death, total death, hospitalization for heart failure, and renal outcome. DPP-4i: dipeptydil-peptidase-4 inhibitors; Pl: placebo; GLP-1RA: glucagon-like peptide-1 receptor agonists; SGLT-2i: sodium-glucose cotransporter-2 inhibitors

As for the single components of MACE, Fig. 2 and Additional file 1: Fig. S4 show that both GLP-1RA and SGLT-2 inhibitors were associated with a lower risk for nonfatal MI as compared with placebo (9% and 13%, respectively), or with DPP-4 inhibitors (10% and 14%, respectively), which was significant for the comparison between SGLT-2 and DPP-4 inhibitors only. The comparison between GLP-1RA and SGLT-2 inhibitors shows no difference in their risk for nonfatal MI (RR = 1.04, 95% CI 0.92–1.19). P-rank scores show that SGLT-2 inhibitors were ranked first in reducing nonfatal MI (90.4%) (Additional file 1: Table S2). As for nonfatal stroke, Fig. 2 and Additional file 1: Fig. S5 show that GLP-1RA were associated with lower risks as compared with placebo (16% lower risk), DPP-4 inhibitors (15% lower risk) and SGLT-2 inhibitors (10% lower risk), but the difference was statistically significant as compared with placebo only. P-rank scores show that GLP-1RA were ranked first in reducing nonfatal MI (95.8%) (Additional file 1: Table S2). As for CV death, Fig. 3 and Additional file 1: Fig. S6 show that both GLP-1RA and SGLT-2 inhibitors were associated with a lower risk (13% and 14%, respectively) as compared with placebo and DPP-4 inhibitors (11% and 12%, respectively), although the difference in risks was significant for SGLT-2 vs DPP-4 inhibitors only. There was no difference in the risk of CV death when GLP-1RA were compared with SGLT-2 inhibitors. P-rank scores show that SGLT-2 inhibitors were ranked first in reducing CV death (84.8%) (Additional file 1: Table S2). The overall heterogeneity levels were 24.4% (95% uncertainty intervals, 0 and 54.9%) for nonfatal MI (moderate), 11.1% (95% uncertainty intervals, 0 and 48.0%) for nonfatal stroke (low) and 15.8% (95% uncertainty intervals, 0 and 49.9%) for CV death (moderate) (Additional file 1: Table S3). The Egger test for these outcomes indicated some asymmetry for CV death only (P = 0.10).

Figure 3 and Additional file 1: Fig. S7 show the results for total death. Both GLP-1RA and SGLT-2 inhibitors were associated with the same lower risk (12%) as compared with placebo, and the same lower risk as compared with DPP-4 inhibitors (13%). The comparison between GLP-1RA and SGLT-2 inhibitors shows no difference in their risk for total death (RR = 1.00, 95% CI 0.91–1.11). P-rank scores show that SGLT-2 inhibitors were ranked first in reducing total death (84.0%) and GLP-1RA were ranked second (81.9%) (Additional file 1: Table S2). The overall heterogeneity for total death was I^2^ = 33.7% (95% uncertainty intervals, 0 and 60.9%) (moderate) (Additional file 1: Table S3). There was some evidence of publication bias (Egger test, P = 0.94), but the trim-and-fill method indicated that this publication bias did not impact the estimate.

Figure 3 and Additional file 1: Fig. S8 show the results for HHF. Both GLP-1RA and SGLT-2 inhibitors were associated with a lower risk (10% and 31%, respectively) as compared with placebo, and an even greater lower risk as compared with DPP-4 inhibitors (15% and 35%, respectively). Moreover, SGLT-2 inhibitors were associated with a lower risk for HHF as compared with GLP-1RA (RR = 0.76, 95% CI 0.68–0.85). P-rank scores show that SGLT-2 inhibitors were ranked first in reducing HHF (100%) (Additional file 1: Table S2). The overall heterogeneity for HHF was I^2^ = 0% (95% uncertainty intervals, 0 and 48.0%) (null) (Additional file 1: Table S3), with no evidence of publication bias (Egger test, P = 0.413).

Figure 3 and Additional file 1: Fig. S9 show the results for the renal outcome which was different among trials (Table 2). SGLT-2 inhibitors were associated with lower risk of renal outcome as compared with placebo (36% lower risk), DPP-4 inhibitors (40% lower risk) and GLP-1RA (22% lower risk). Moreover, GLP-1RA were associated with lower risk for renal outcomes as compared with either placebo (18% lower risk) or DPP-4 inhibitors (22% lower risk). P-rank scores show that SGLT-2 inhibitors were ranked first in reducing renal outcome (100%) (Additional file 1: Table S2). The overall heterogeneity for renal outcome was I^2^ = 42.3% (95% uncertainty intervals, 0 and 68.0%) (moderate) (Additional file 1: Table S3) with no evidence of publication bias.

**Table 2:** The composite renal outcome in CVOTs

SAVOR-TIMI 53	Doubling of creatinine level, initiation of dialysis, renal transplantation, or creatinine > 6.0 mg/dl
EXAMINE	Renal dialysis
TECOS	Renal failure
CARMELINA	End stage renal disease, death due to kidney failure, or sustained decrease of ≥ 40% in eGFR from baseline
ELIXA*	Doubling of serum creatinine
LEADER	New onset, persistent macroalbuminuria, persistent doubling of serum creatinine along with an eGRF < 45 ml/min/1.73 m^2^, need of renal-replacement therapy or death from kidney disease
SUSTAIN-6	Persistent macroalbuminuria, persistent doubling of serum creatinine along with a creatinine clearance < 45 ml/min/1.73 m^2^, need of continuous renal-replacement therapy
EXSCEL	New-onset macroalbuminuria, 40% reduction of eGFR, initiation of renal replacement therapy, and death from renal causes
HARMONY	Change in eGFR, worsening renal function (safety outcomes)
REWIND	New-onset macroalbuminuria (UACR > 33.9 mg/mmol), ≥ 30% decline in eGRF, or new renal replacement therapy comprising dialysis or renal transplantation
PIONEER 6	Not reported
AMPLITUDE-O	A decrease in the eGFR of ≥ 40% for ≥ 30 days, end-stage kidney disease (defined as dialysis for ≥ 90 days, kidney transplantation, or an eGFR of < 15 ml per minute per 1.73 m2 for ≥ 30 days), or death from any cause
EMPA-REG	A doubling of the serum creatinine level, the initiation of renal-replacement therapy, or death from renal disease
CANVAS	≥ 40% reduction in eGFR, renal-replacement therapy, or renal death
DECLARE	≥ 40% decrease in eGFR to < 60 ml/min/1.73 m^2^, ESRD, or death from renal cause
DAPA-HF	≥ 50% sustained decline eGFR or end-stage renal disease or renal death
CREDENCE	End-stage kidney disease, doubling of serum creatinine level, or renal death
VERTIS-CV	Death from renal causes, renal replacement therapy, or doubling of the serum creatinine level
DAPA-CKD	Sustained decline in the estimated GFR of at least 50%, end-stage kidney disease, or death from renal causes
EMPEROR-R	Chronic dialysis or renal transplantation or a profound, sustained reduction in the estimated GFR
SCORED	First occurrence of a sustained decrease of ≥ 50% in the eGFR from baseline for ≥ 30 days, long-term dialysis, renal transplantation, or sustained eGFR of < 15 ml/min/1.73 m^2^ for ≥ 30 days
SOLOIST-WHF	Not reported
EMPEROR-P	The rate of decline in the eGFR during double-blind treatment

*As indicated in a post-hoc analysis (Lancet Diabetes Endocrinol. 2018;6:859–869)

**Fig. 4:**
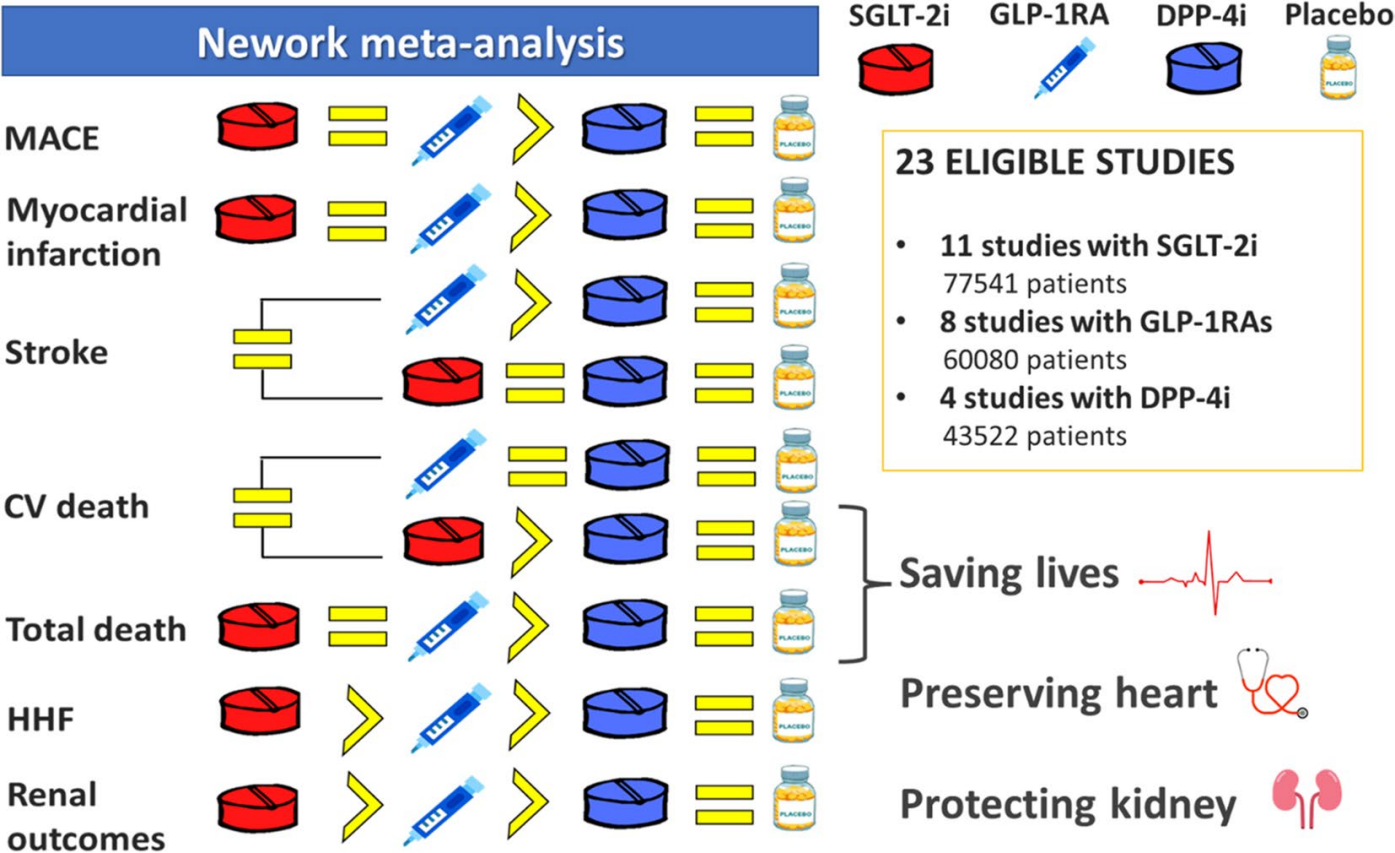
Summary “at glance” of the network meta-analysis comparing the effects of SGLT-2 inhibitors, GLP-1RA and DPP-4 inhibitors on cardiorenal outcomes. The symbol = indicates non significantly different; the symbol > indicates significantly different

Figure 4 and Additional file 1: Table S5 show the overall effects of the three classes of drugs on cardiorenal endpoints by the network meta-analysis using the frequentist approach.

## Discussion

The present network meta-analysis included the most recent published CVOTs, thus providing the most contemporary assessment of the total available evidence for DPP-4 inhibitors, GLP-1RA and SGLT-2 inhibitors and their cardiorenal outcomes. The results of our analysis confirm the lack of any benefit by DPP-4 inhibitors on cardiorenal outcomes in people with type 2 diabetes [22]. Although past meta-analyses reported substantial reductions in MACE with DPP-4 inhibitors [23], their conclusions were based on studies with small sample sizes and limited numbers of cardiovascular events, which have not been confirmed by the four CVOTs specifically designed to assess the CV safety of DPP-4 inhibitors. Our analysis also confirms that GLP-1RA, when compared with placebo, reduce the risk of MACE, total death, HHF, and the composite renal outcome [12]. Finally, when compared with placebo, SGLT-2 inhibitors are associated with a robust reduction of hospitalization for HHF and renal outcome, and a moderate reduction of CV and total death, and MACE [13].

As for the network meta-analysis, Fig. 4 shows an “at glance” summary of the results obtained. According to our data, SGLT-2 inhibitors show the highest probability to be superior to GLP-1RA and DPP-4 inhibitors in terms of HHF and the composite renal outcome, while GLP1-RA are superior to DPP-4 inhibitors for both outcomes. These results are based on 22 comparisons for HHF and 18 comparisons for the renal outcome. As for mortality, both SGLT-2 inhibitors and GLP-1RA are similar in reducing both total and CV deaths, but SGLT-2 inhibitors only are superior to DPP-4 inhibitors for both deaths, whereas GLP-1RA are superior to DPP-4 inhibitors for total death, but equal for CV death. These results are based on all 23 comparisons. As for nonfatal stroke, GLP-1 RA are the only drug class that significantly reduces nonfatal stroke, as compared with placebo, without any difference among the three classes of drugs. These results, as those for nonfatal MI and MACE, are based on 18 comparisons. As for nonfatal MI, both SGLT-2 inhibitors and GLP-1RA are similar, but SGLT-2 inhibitors only are superior to DPP-4 inhibitors. Finally, for the primary endpoint MACE, both SGLT-2 inhibitors and GLP-1RA are similar and both are superior to DPP-4 inhibitors. For the first time, clinicians have the option to save lives with antidiabetic drugs in certain groups of patients with type 2 diabetes.

The inclusion in our network analysis of the AMPLITUDE-O and EMPEROR-P data have modified the conclusions of the most recent similar analysis [15] indicating that SGLT-2 inhibitors increased the risk of stroke as compared with GLP-1RA and that GLP-1RA are superior to DPP-4 inhibitors as for nonfatal MI. Although EMPEROR-R enrolled patients with and without type 2 diabetes, the primary outcome of the trial (a composite of CV death and hospitalization for HF) was almost identical in both groups, suggesting that the different population didn’t affect the results, at least for the primary outcome.

Owing to intrinsic limitations of network meta-analysis, we avoided to analyze the effects of these drugs in several subgroups—e.g., as stratified by age or the presence of CV disease or type 2 diabetes—to avoid type 1 error due to the many subgroup analyses. Moreover, these analyses have already been done in conventional meta-analyses, showing for SGLT-2 inhibitors the lack of significant difference in the reduced risk of the composite outcome (CV death + HHF) in patients with or without type 2 diabetes or in subjects of 65 years of age or younger vs those older than 65 years of age [13]. Moreover, GLP-1RA reduced the risk of MACE by 14% in the overall diabetic population, with an apparent greater effect in patients with established CV disease (16% vs 6% reduction, respectively), although the lack of significant interaction between subgroups does not allow to separate them [12].

As evidence of the efficacy of SGLT-2 inhibitors continued to grow, trials on these drugs have expanded their study populations from diabetes patients only to also include patients with HF or CKD in the absence of diabetes. On the basis of results of the most recent CVOTs (DAPA-HF, DAPA-CKD, EMPEROR-R), the FDA has approved dapagliflozin (2020) and empagliflozin (2021) to reduce risk for CV death and HF hospitalization in adults with HF and reduced ejection fraction regardless of whether they have diabetes [24, 25]. FDA has also approved dapagliflozin for treatment for CKD (2021) [26] and has given priority review for empagliflozin to potentially treat heart failure not associated with left ventricular ejection fraction [27]. SGLT-2 inhibitors and GLP-1RA should, therefore, be considered evidence-based treatments for patients with type 2 diabetes after metformin. In theory, SGLT-2 inhibitors should be considered before GLP-1RA because of the reduction in both CV and total deaths associated with their use. In practice, it seems better to tailor the choice to different patients, depending on the preference of the route of administration (oral vs injectable), the presence of intolerance or side-effects, and contraindications; in these cases, one can be switched to another, or they can both be given, if the glycemic target is not attained.

To confirm that CVOTs findings are consistent in more diverse populations reflective of patients in the clinical practice, we may need to look beyond clinical trials to real-world evidence studies. In a meta-analysis evaluating the real-world effect of SGLT-2 inhibitors on cardiovascular outcome in patients with type 2 diabetes, Li et al. [28] included fourteen trials enrolling 3,157,259 patients. They found that the predominant impact of SGLT-2 inhibitors is on cardiovascular outcome was driven predominantly by reduction in MACE, total death, HHF, MI, stroke, and CV death. These results were even greater than those recorded in CVOTs. Quite similar results, although of lesser extent and for lesser outcomes, have been observed with GLP-1RA [29].

Our network meta-analysis has certain limitations. Firstly, there was no head-to-head CVOT directly comparing these antidiabetic drug classes; therefore, the comparative effects were generated with indirect evidence, and caution must be exercised when interpreting data from indirect comparison of CVOTs. Moreover, different drugs were used within each drug class, and there could be within-class differences. However, there are on the horizon no cardiorenal outcome studies comparing SGLT-2 inhibitors with GLP-1RA, so it is likely that we must rely on indirect comparisons. Secondly, as for most meta-analyses, we did not have patient-level data, limiting the scope for adjustments. Finally, there were differences in trial designs, patient characteristics, background therapy, and endpoint definitions. However, and despite these limitations, SGLT-2 inhibitors or a GLP-1RA with proven CV disease benefit are recommended in patients with type 2 diabetes and CV disease, established kidney disease, or HF [7–10].

Strengths of the present meta-analysis are the inclusion of all CVOTs published by 10 December 2021, the very large number of participants, the high quality of the trials which minimizes the risk of bias, and the absence of significant heterogeneity in most analyses, which ranged from absent to low or moderate. The clinical relevance of these results seems also highlighted by the evidence that for some outcomes the clinical benefit is consistent irrespective of the presence of type 2 diabetes [13], advanced age [13, 30], and the background cardiorenal disease [12, 15, 31]. Accordingly, people with type 2 diabetes, cardiovascular disease, heart failure, or chronic kidney disease should be treated appropriately with an SGLT-2 inhibitor or GLP-1RA, even because more adults with type 2 diabetes in the US have suboptimal glycemic control now compared to 10 years ago, associated with a resurgence in vascular diabetic complications [32].

## Conclusions

In summary, SGLT-2 inhibitors are superior in reducing cardiovascular death, hospitalization for HF, and renal events among the new antidiabetic drug classes. Both SGLT-2 inhibitors and GLP-1 RA can reduce MACE. In terms of cardiovascular and renal outcome, DPP-4 inhibitors are comparable to placebo and are inferior to the other two drug classes. SGLT-2 inhibitors and GLP-1RA, should be the preferred treatment for patients with type 2 diabetes and cardiorenal diseases after metformin. It is not by chance that the most recent clinical guidelines [10] continue to advocate the use of antidiabetic drugs that have been proven to reduce cardiovascular events and mortality.

## Supplementary Information

**Additional file 1:** **Figure S1. **Process of studies’ selection. **Table S1. **Summary of risk of bias assessment. **Figure S2. **Cochrane risk of bias (graph) for the 23 trials. **Figure S3.** Risk ratios (RR) for MACE estimates according to direct (DPP-4 inhibitors, GLP-1RA and SGLT-2 inhibitors against placebo) and indirect (DPP-4 inhibitors vs GLP-1RA or SGLT-2 inhibitors, and GLP-1RA vs SGLT-2 inhibitors) comparisons. **Table S2**. Ranking of treatments by P-score value for all outcomes. **Table S3**. Overall heterogeneity levels for each of the seven outcomes. **Figure S4.** Risk ratios (RR) for nonfatal myocardial infarction estimates according to direct (DPP-4 inhibitors, GLP-1RA and SGLT-2 inhibitors against placebo) and indirect (DPP-4 inhibitors vs GLP-1RA or SGLT-2 inhibitors, and GLP-1RA vs SGLT-2 inhibitors) comparisons. **Figure S5.** Risk ratios (RR) for nonfatal stroke estimates according to direct (DPP-4 inhibitors, GLP-1RA and SGLT-2 inhibitors against placebo) and indirect (DPP-4 inhibitors vs GLP-1RA or SGLT-2 inhibitors, and GLP-1RA vs SGLT-2 inhibitors) comparisons. **Figure S6.** Risk ratios (RR) for cardiovascular death estimates according to direct (DPP-4 inhibitors, GLP-1RA and SGLT-2 inhibitors against placebo) and indirect (DPP-4 inhibitors vs GLP-1RA or SGLT-2 inhibitors, and GLP-1RA vs SGLT-2 inhibitors) comparisons. **Figure S7.** Risk ratios (RR) for total death estimates according to direct (DPP-4 inhibitors, GLP-1RA and SGLT-2 inhibitors against placebo) and indirect (DPP-4 inhibitors vs GLP-1RA or SGLT-2 inhibitors, and GLP-1RA vs SGLT-2 inhibitors) comparisons. **Figure S8.** Risk ratios (RR) for hospitalization for heart failure estimates according to direct (DPP-4 inhibitors, GLP-1RA and SGLT-2 inhibitors against placebo) and indirect (DPP-4 inhibitors vs GLP-1RA or SGLT-2 inhibitors, and GLP-1RA vs SGLT-2 inhibitors) comparisons. **Figure S9.** Risk ratios (RR) for the renal outcome estimates according to direct (DPP-4 inhibitors, GLP-1RA and SGLT-2 inhibitors against placebo) and indirect (DPP-4 inhibitors vs GLP-1RA or SGLT-2 inhibitors, and GLP-1RA vs SGLT-2 inhibitors) comparisons. **Table S4.** Effects of novel antidiabetic drugs on cardiorenal endpoints established by network meta-analysis using a frequentist approach. PRISMA checklist.

## Data Availability

All data generated or analyzed during this study are included in this published article and in its Additional file.

## Abbreviations

CVOTs: Cardiovascular outcome trials
DPP-4 inhibitors: Dipeptidyl peptidase-4 inhibitors
GLP-1RA: Glucagon-like peptide-1 receptor agonists
SGLT-2 inhibitors: Sodium-glucose cotransporter 2 inhibitors
MACE: Major cardiovascular events
RR: Risk ratio
HHF: Hospitalization for heart failure
