# Supplementary material for: The effect of DPP-4 inhibitors, GLP-1 receptor agonists and SGLT-2 inhibitors on cardiorenal outcomes: a network meta-analysis of 23 CVOTs
Source: Cardiovasc Diabetol. 2022 Mar 16;21:42. doi: 10.1186/s12933-022-01474-z (PMC8925229; doi:10.1186/s12933-022-01474-z)
Supplement: Supplementary file 1 — Additional file 1: Figure S1. Process of studies’ selection. Table S1. Summary of risk of bias assessment. Figure S2. Cochrane risk of bias (graph) for the 23 trials. Figure S3. Risk ratios (RR) for MACE estimates according to direct (DPP-4 inhibitors, GLP-1RA and SGLT-2 inhibitors against placebo) and indirect (DPP-4 inhibitors vs GLP-1RA or SGLT-2 inhibitors, and GLP-1RA vs SGLT-2 inhibitors) comparisons. Table S2. Ranking of treatments by P-score value for all outcomes. Table S3. Overall heterogeneity levels for each of the seven outcomes. Figure S4. Risk ratios (RR) for nonfatal myocardial infarction estimates according to direct (DPP-4 inhibitors, GLP-1RA and SGLT-2 inhibitors against placebo) and indirect (DPP-4 inhibitors vs GLP-1RA or SGLT-2 inhibitors, and GLP-1RA vs SGLT-2 inhibitors) comparisons. Figure S5. Risk ratios (RR) for nonfatal stroke estimates according to direct (DPP-4 inhibitors, GLP-1RA and SGLT-2 inhibitors against placebo) and indirect (DPP-4 inhibitors vs GLP-1RA or SGLT-2 inhibitors, and GLP-1RA vs SGLT-2 inhibitors) comparisons. Figure S6. Risk ratios (RR) for cardiovascular death estimates according to direct (DPP-4 inhibitors, GLP-1RA and SGLT-2 inhibitors against placebo) and indirect (DPP-4 inhibitors vs GLP-1RA or SGLT-2 inhibitors, and GLP-1RA vs SGLT-2 inhibitors) comparisons. Figure S7. Risk ratios (RR) for total death estimates according to direct (DPP-4 inhibitors, GLP-1RA and SGLT-2 inhibitors against placebo) and indirect (DPP-4 inhibitors vs GLP-1RA or SGLT-2 inhibitors, and GLP-1RA vs SGLT-2 inhibitors) comparisons. Figure S8. Risk ratios (RR) for hospitalization for heart failure estimates according to direct (DPP-4 inhibitors, GLP-1RA and SGLT-2 inhibitors against placebo) and indirect (DPP-4 inhibitors vs GLP-1RA or SGLT-2 inhibitors, and GLP-1RA vs SGLT-2 inhibitors) comparisons. Figure S9. Risk ratios (RR) for the renal outcome estimates according to direct (DPP-4 inhibitors, GLP-1RA and SGLT-2 inhibitors against place [file 12933_2022_1474_MOESM1_ESM.docx]

**The effect of DPP-4 inhibitors, GLP-1 receptor agonists and SGLT-2 inhibitors on cardiorenal outcomes: a net-work meta-analysis of 23 CVOTs**

Dario Giugliano, Miriam Longo, Simona Signoriello, Maria Ida Maiorino, Bruno Solerte, Paolo Chiodini, Katherine Esposito

**Supplemental Material (pag. 1-21)**

*Supplementary Figure S1……………………………………………………………………………………………. pag. 2*

*References of 23 CVOTs included in the network analysis ………………………………………… pag. 3*

*Supplementary Table S1……………………………………………………………………………………………… pag. 5*

*Supplementary Figure S2 …………………………………………………………………………………………… pag. 6*

*Supplementary Figure S3 …………………………………………………………………………………………… pag. 7*

*Supplementary Tables 2 and 3 ……………………………………………………………………………………… pag. 8*

*Supplementary Figure S4 ……………………………………………………………………………………………. pag. 9*

*Supplementary Figure S5 ……………………………………………………………………………………………. pag. 10*

*Supplementary Figure S6 ……………………………………………………………………………………………. pag. 11*

*Supplementary Figure S7 …………………………………………………………………………………………… pag. 12*

*Supplementary Figure S8 ………………………………………………………………………………………….. pag.13*

*Supplementary Figure S9 ………………………………………………………………………………………… pag.14*

*Supplementary Table S4 ……………………………………………………………………………………………. pag. 15*

*PRISMA Statement …………………………………………………………………………………………………….. pag. 17*

**Screening**

**Included**

**Eligibility**

**Identification**

Titles/abstracts assessed for

eligibility

(n = 433)

Duplicate excluded

(n = 369)

Full-text article assessed for eligibility

(n = 54)

Records identified through database search (n = 802)

Excluded (n=379)

-Design of study (50)

-Commentary or editorials (321)

-No full-text report (8)

Excluded (n=31)

-No MACE or renal outcome

reported

-No relevant secondary analysis

Trials included

(n = 23)

**Figure S1.** Process of studies’ selection.

References for the 23 CVOTs (4 studies with DPP-4 inhibitors, 8 studies with GLP-1RA, and 11 studies with SGLT-2 inhibitors)

1. Scirica BM, Bhatt DL, Braunwald E, et al. SAVOR-TIMI 53 steering committee and investigators. Saxagliptin and cardiovascular outcomes in patients with type 2 diabetes mellitus. N Engl J Med. 2013; 369:1317-1326.
2. White WB, Cannon CP, Heller SR, et al. Alogliptin after acute coronary syndrome in patients with type 2 diabetes. N Engl J Med. 2013;369:1327-1335.
3. Green JB, Bethel MA, Armstrong PW. et al. Effect of sitagliptin on cardiovascular outcomes in type 2 diabetes. N Engl J Med. 2015;373:232-242.
4. Rosenstock J, Perkovic V, Johansen OE, et al. Effect of linagliptin vs placebo on major cardiovascular events in adults with type 2 diabetes and high cardiovascular and renal risk. The CARMELINA randomized clinical trial. JAMA. 2019;321:69-79.
5. Pfeffer MA, Claggett B, Diaz R, et al. Lixisenatide in patients with type 2 diabetes and acute coronary syndrome. N Engl J Med. 2015;373:2247-2257.
6. Marso SP, Daniels GH, Brown-Frandsen K, et al. Liraglutide and cardiovascular outcomes in type 2 diabetes. N Engl J Med. 2016;375:311-322.
7. Marso SP, Bain SC, Consoli A, et al. Semaglutide and cardiovascular outcomes in patients with type 2 diabetes. N Engl J Med. 2016;375:1834-1844.
8. Holman RR, Bethel MA, Mentz RJ, et al. Effects of once-weekly exenatide on cardiovascular outcomes in type 2 diabetes. N Engl JMed. 2017;377:1228-1239.
9. Hernandez AF, Green JB, Janmohamed S, et al. Albiglutide and cardiovascular outcomes in patients with type 2 diabetes and cardiovascular disease (Harmony outcomes): a double-blind, randomized placebo-controlled trial. Lancet. 2018;392:1519-1529.
10. Gerstein HC, Colhoun HM, Dagenais GR, et al. Dulaglutide and cardiovascular outcomes in type 2 diabetes (REWIND): a doubleblind, randomised placebo-controlled trial. Lancet. 2019;394:121-130.
11. Husain M, Birkenfeld AL, Donsmark M, et al. For the PIONEER 6 investigators. Oral semaglutide and cardiovascular outcomes in patients with type 2 diabetes. N Engl J Med. 2019;381:841-851.
12. Gerstein HC, Sattar N, Rosenstock J, et al; for the AMPLITUDE-O Trial Investigators. Cardiovascular and renal outcomes with efpeglenatide in type 2 diabetes. New Engl J Med. 2021;385(10):896-907.
13. Zinman B, Wanner C, Lachin JM, et al. Empagliflozin, cardiovascular outcomes, and mortality in type 2 diabetes. N Engl J Med. 2015;373:2117-2128
14. Neal B, Perkovic V, Mahaffey KW, et al. Canagliflozin and cardiovascular and renal events in type 2 diabetes. N Engl J Med. 2017;377:644-657
15. Wiviott SD, Raz I, Bonaca MP, et al. Dapagliflozin and cardiovascular outcomes in type 2 diabetes. N Engl J Med. 2019;380:347-357
16. Perkovic V, Jardine MJ, Neal B, et al. Canagliflozin and renal outcomes in type 2 diabetes and nephropathy. N Engl J Med. 2019;380:2295-2306
17. McMurray JJV, Solomon SD, Inzucchi SE, et al. Dapagliflozin in patients with heart failure and reduced ejection fraction. N Engl J Med. 2019;381:1995-2008
18. Heerspink HJL, Stefánsson BV, Correa-Rotter R, et al. Dapagliflozin in patients with chronic kidney disease. N Engl J Med. 2020; 383:1436-1446
19. Cannon CP, Pratley R, Dagogo-Jack S, et al. Cardiovascular outcomes with ertugliflozin in type 2 diabetes. N Engl J Med. 2020;383(15):1425-1435
20. Packer M, Anker SD, Butler J, et al. Cardiovascular and renal outcomes with empagliflozin in heart failure. N Engl J Med. 2020; 383:1413-1424
21. Bhatt DL, Szarek M, Pitt B, Cannon CP, et al. Sotagliflozin in patients with diabetes and chronic kidney disease. N Engl J Med. 2021;384(2):129-139
22. Bhatt DL, Szarek M, Steg PG, et al. Sotagliflozin in patients with diabetes and recent worsening heart failure. N Engl J Med. 2021;384:117-128.
23. Anker SD, Butler J, Filippatos G, et al. Empagliflozin in heart failure with a preserved ejection fraction. N Engl J Med. 385:1451-1461.

| **Table S1.** Summary of risk of bias assessment | | | | |  |  |  |
| --- | --- | --- | --- | --- | --- | --- | --- |
| Study ID | Random sequence generation* | | Allocation concealment* | Blinding of participants and personnel° | Blinding of outcome assessment° | Incomplete outcome data° | Selective reporting° |
| SAVOR-TIMI 53, 2013 | L | | L | L | L | L | L |
| EXAMINE, 2013 | L | | L | L | L | L | L |
| TECOS, 2015 | L | | L | L | L | L | L |
| CARMELINA, 2019 | L | | L | L | L | L | L |
| ELIXA, 2015 | L | | L | L | L | L | L |
| LEADER, 2016 | L | | L | L | L | L | L |
| SUSTAIN-6, 2016 | L | | L | L | L | L | L |
| EXSCEL, 2017 | L | | L | L | L | L | L |
| HARMONY, 2018 | L | | L | L | L | L | L |
| REWIND, 2019 | L | | L | L | L | L | L |
| PIONEER 6, 2019 | L | | L | L | L | L | L |
| AMPLITUDE-O, 2021 | L | | L | L | L | L | L |
| EMPA-REG, 2015 | L | | L | L | L | L | L |
| CANVAS, 2017 | L | | L | L | L | L | L |
| DECLARE, 2019 | L | | L | L | L | L | L |
| CREDENCE, 2019 | L | | L | L | L | L | L |
| DAPA-HF, 2019 | L | | L | L | L | L | L |
| DAPA-CKD, 2020 | L | | L | L | L | L | L |
| VERTIS-CV, 2020 | L | | L | L | L | L | L |
| EMPEROR-R, 2020 | L | | L | L | L | L | L |
| SCORED, 2021 | L | | L | L | L | L | L |
| SOLOIST-WHF, 2021 | L | | L | L | L | L | L |
| EMPEROR-P, 2021 | L | | L | L | L | L | L |
| L= low risk of bias; U= unclear risk of bias; H= high risk of bias | | | | |  |  |  |
| *Risk of bias assessment for random sequence generation and allocation concealment is | | | | | | |  |
| performed at the study level. | | |  |  |  |  |  |
| °Risk of bias assessment for blinding of participants and personnel, blinding of outcome | | | | | | |  |
| assessment, incomplete outcome data, and selective reporting are for the primary outcome. | | | | | | |  |
|  | |  |  |  |  |  |  |


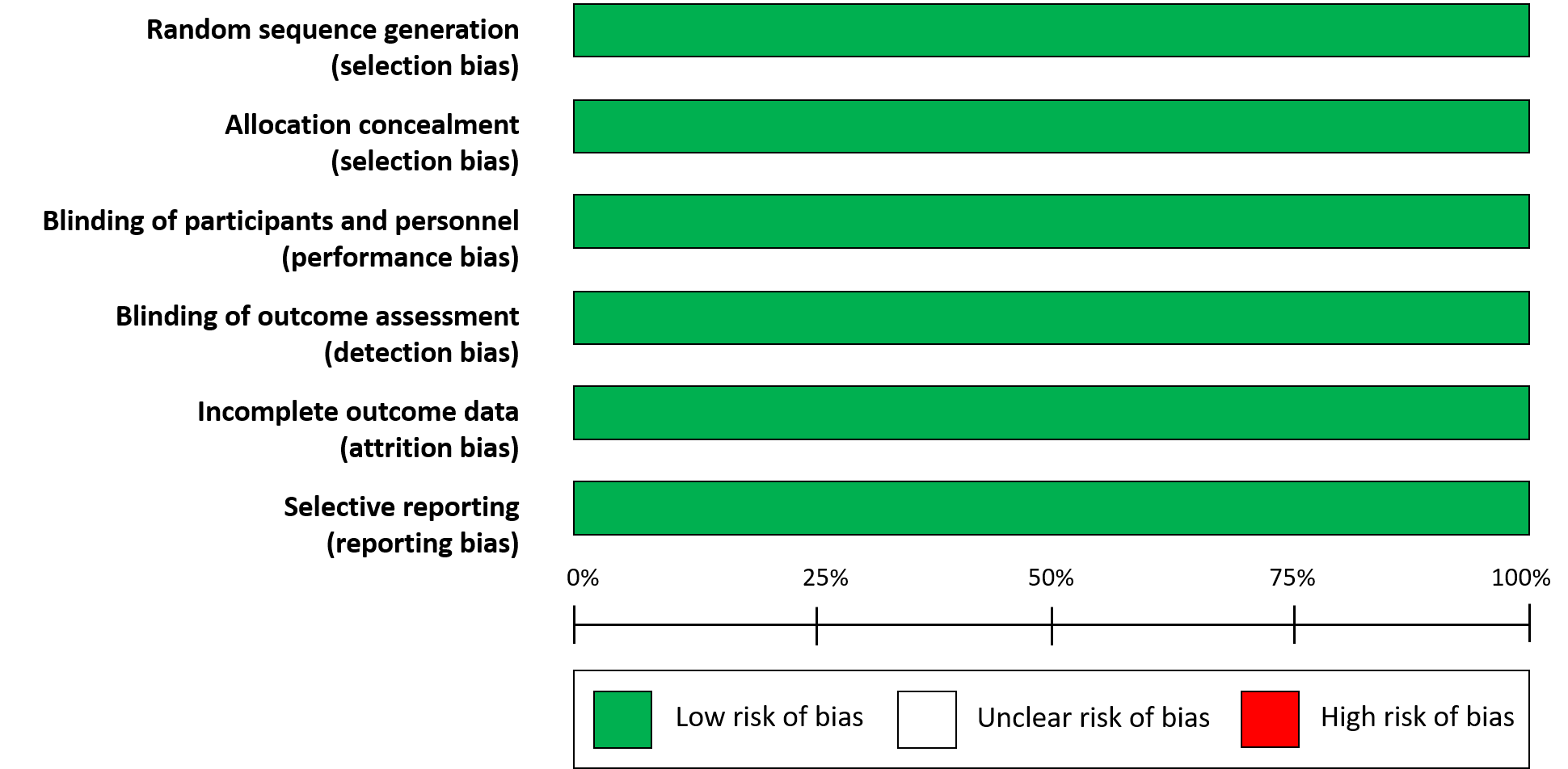


**Figure S2.** Cochrane risk of bias (graph) for the 23 trials


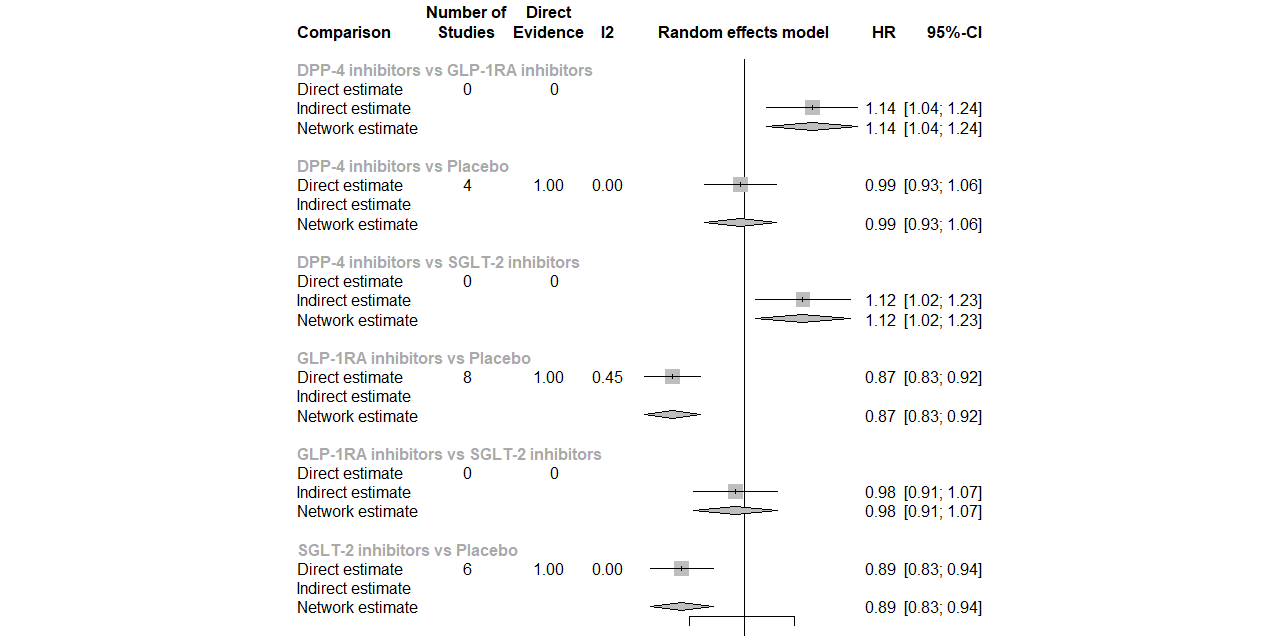


RR

RRRR

**Figure S3.** Risk ratios (RR) for MACE estimates according to direct (DPP-4 inhibitors, GLP-1RA and SGLT-2 inhibitors against placebo) and indirect (DPP-4 inhibitors vs GLP-1RA or SGLT-2 inhibitors, and GLP-1RA vs SGLT-2 inhibitors) comparisons.

**Table S2**. Ranking of treatments by P-score value for all outcomes.

A higher value of P-score indicate the higher probability to be the best in the four comparisons.

**Table S3**. Overall heterogeneity levels for each of the seven outcomes.

|  | I^2^ (95% uncertainty intervals) Q P value |
| --- | --- |
| MACE | 15.3% [0.0%; 52.3%] 17.7 0.278 |
| HHF | 0.0% [0.0%; 48.0%] 14.4 0.763 |
| CV death | 15.8% [0.0%; 49.9%] 23.7 0.254 |
| Nonfatal MI | 24.4% [0.0%; 54.9%] 19.8 0.178 |
| Nonfatal stroke | 11.1% [0.0%; 48.4%] 16.9 0.327 |
| Total death | 33.7% [0.0%; 60.9%] 30.1 0.068 |
| Renal outcome | 42.3% [0.0%; 68.0%] 26.0 0.038 |


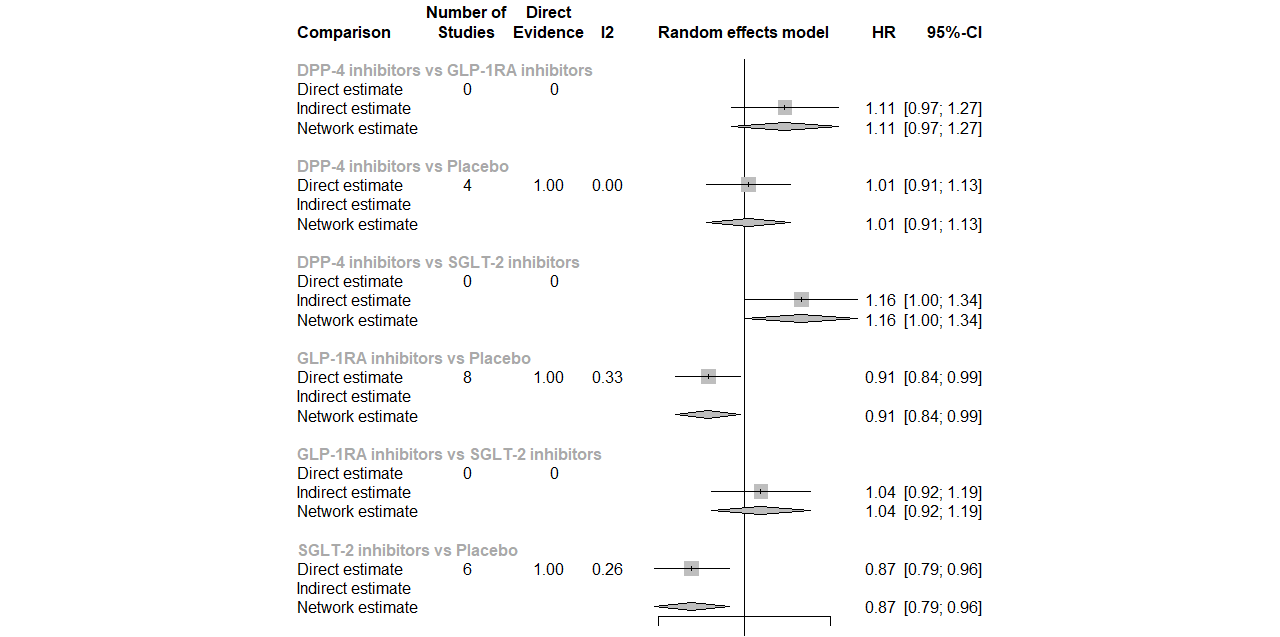


RR

**Figure S4.** Risk ratios (RR) for nonfatal myocardial infarction estimates according to direct (DPP-4 inhibitors, GLP-1RA and SGLT-2 inhibitors against placebo) and indirect (DPP-4 inhibitors vs GLP-1RA or SGLT-2 inhibitors, and GLP-1RA vs SGLT-2 inhibitors) comparisons.


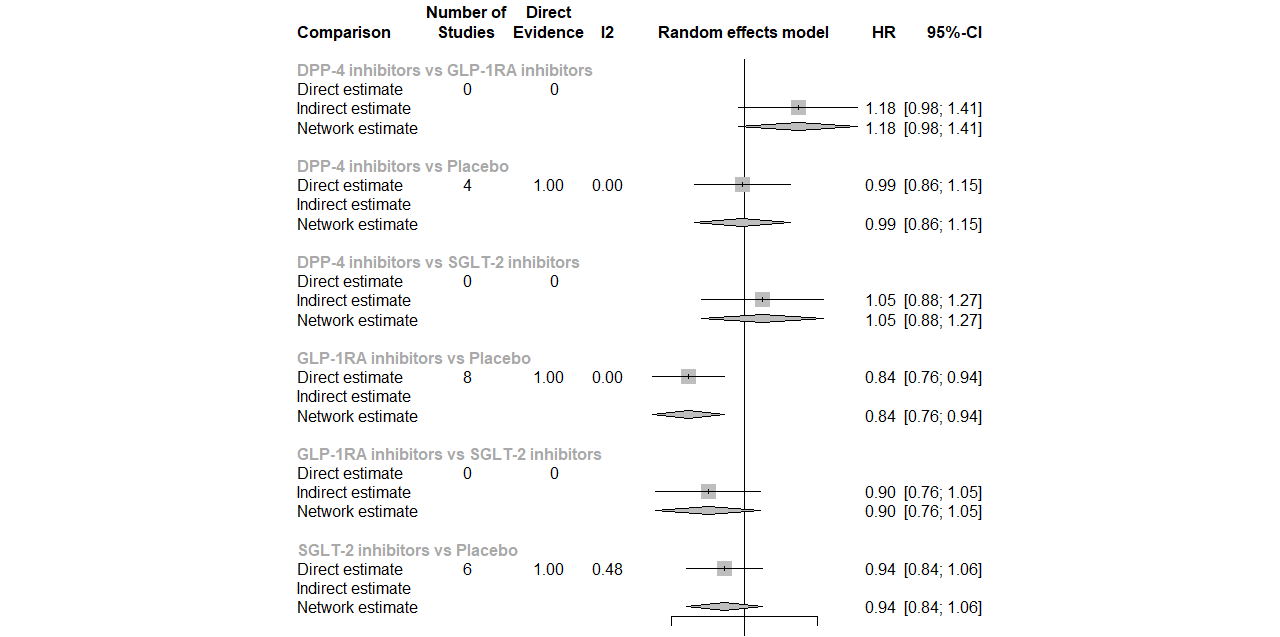


RR

**Figure S5.** Risk ratios (RR) for nonfatal stroke estimates according to direct (DPP-4 inhibitors, GLP-1RA and SGLT-2 inhibitors against placebo) and indirect (DPP-4 inhibitors vs GLP-1RA or SGLT-2 inhibitors, and GLP-1RA vs SGLT-2 inhibitors) comparisons.


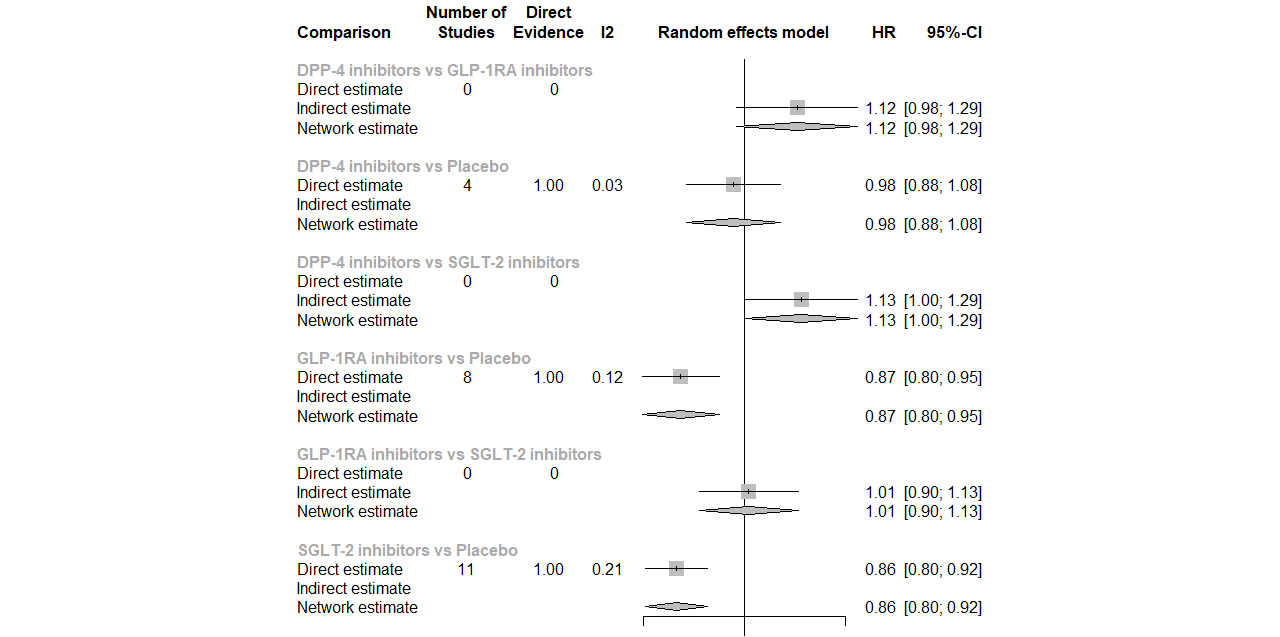


RR

**Figure S6.** Risk ratios (RR) for cardiovascular death estimates according to direct (DPP-4 inhibitors, GLP-1RA and SGLT-2 inhibitors against placebo) and indirect (DPP-4 inhibitors vs GLP-1RA or SGLT-2 inhibitors, and GLP-1RA vs SGLT-2 inhibitors) comparisons.


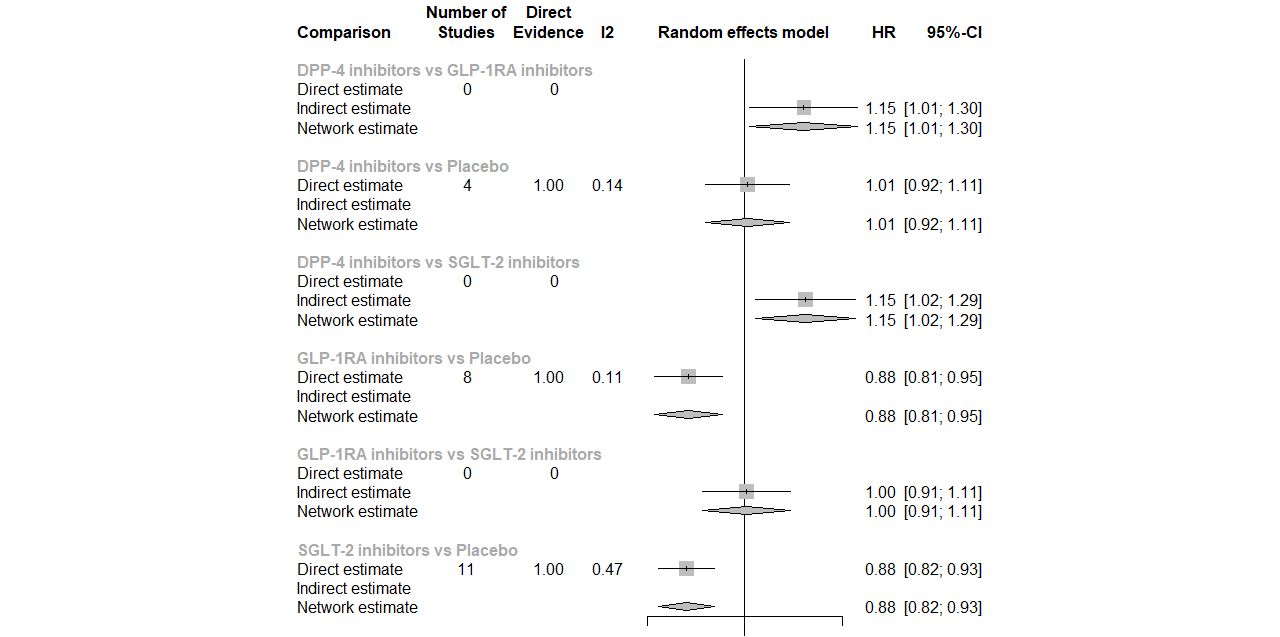


RR

**Figure S7.** Risk ratios (RR) for total death estimates according to direct (DPP-4 inhibitors, GLP-1RA and SGLT-2 inhibitors against placebo) and indirect (DPP-4 inhibitors vs GLP-1RA or SGLT-2 inhibitors, and GLP-1RA vs SGLT-2 inhibitors) comparisons.


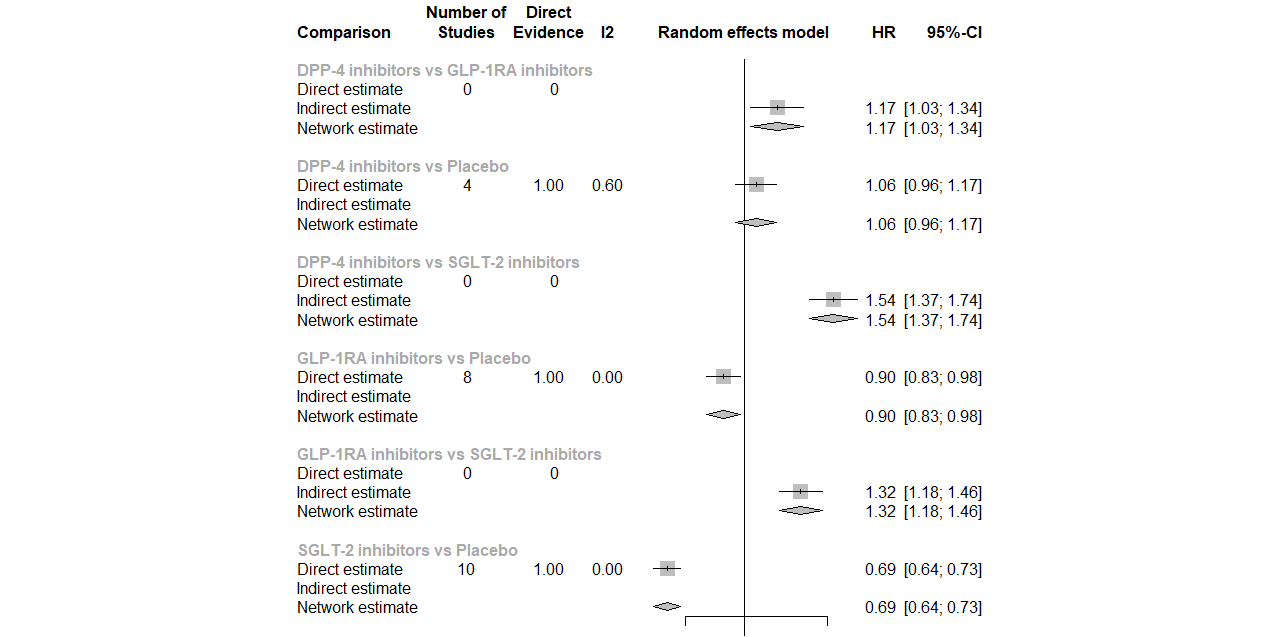


RR

**Figure S8.** Risk ratios (RR) for hospitalization for heart failure estimates according to direct (DPP-4 inhibitors, GLP-1RA and SGLT-2 inhibitors against placebo) and indirect (DPP-4 inhibitors vs GLP-1RA or SGLT-2 inhibitors, and GLP-1RA vs SGLT-2 inhibitors) comparisons.


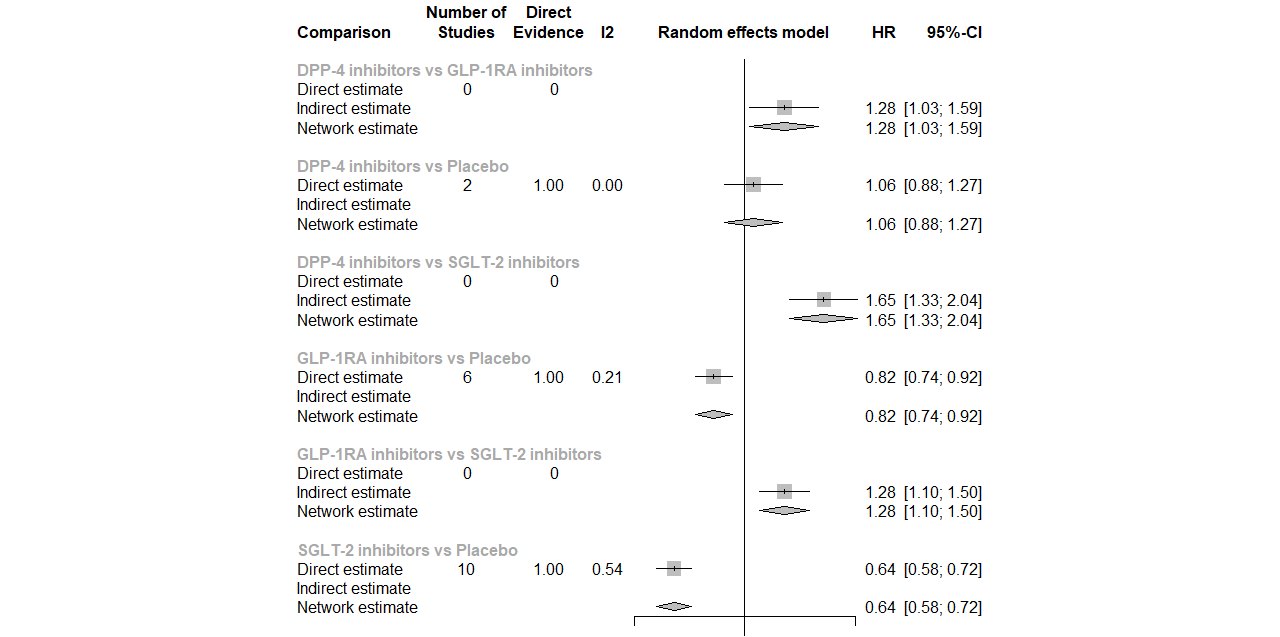


RR

**Figure S9.** Risk ratios (RR) for the renal outcome estimates according to direct (DPP-4 inhibitors, GLP-1RA and SGLT-2 inhibitors against placebo) and indirect (DPP-4 inhibitors vs GLP-1RA or SGLT-2 inhibitors, and GLP-1RA vs SGLT-2 inhibitors) comparisons.

Table S4. Effects of novel antidiabetic drugs on cardiorenal endpoints established by network meta-analysis using a

frequentist approach.

|  | **GLP-1RA** |  | **SGLT-2i** |  | **DPP-4i** |  | **Placebo** |  |
| --- | --- | --- | --- | --- | --- | --- | --- | --- |
| **MACE** |  |  |  |  |  |  |  |  |
| GLP-1RA | 1.0 |  | 1.02 (0.93-1.09) |  | *1.14 (1.04 - 1.23)* |  | *1.15 (1.08-1.20)* |  |
| SGLT-2i | 0.98 (0.91-1.07) |  | 1.0 |  | *1.12 (1.02-1.23)* |  | *1.12 (1.06-1.20)* |  |
| DPP-4i | *0.88 (0.81-0.96)* |  | *0.89 (0.81-0.98)* |  | 1.0 |  | 0.99 (0.93-1.06) |  |
| Placebo | *0.87 (0.83-0.92*) |  | *0.89 (0.83-0.94)* |  | 1.01 (0.94-1.07) |  | 1.0 |  |
| **Nonfatal MI** |  |  |  |  |  |  |  |  |
| GLP-1RA | 1.0 |  | 0.96 (0.84-1.09) |  | 1.11 (0.97-1.28) |  | *1.09 (1.01-1.19)* |  |
| SGLT-2i | 1.04 (0.92-1.19) |  | 1.0 |  | 1.16 (1.0-1.33)  P value= 0.049 |  | *1.15 (1.04-1.27)* |  |
| DPP-4i | 0.90 (0.78-1.03) |  | 0.86 (0.75-1.0)  P value = 0.049 |  | 1.0 |  | 1.01 (0.91-1.13) |  |
| Placebo | *0.91 (0.84-0.99)* |  | *0.87 (0.79-0.96)* |  | 1.01 (0.91-1.13) |  | 1.0 |  |
| **Nonfatal STROKE** |  |  |  |  |  |  |  |  |
| GLP-1RA | 1.0 |  | 1.11 (0.95-1.32) |  | 1.18 (0.98-1.41) |  | *1.19 (1.06-1.31)* |  |
| SGLT-2i | 0.90 (0.76-1.05) |  | 1.0 |  | 1.05 (0.88-1.27) |  | 1.06 (0.94-1.19) |  |
| DPP-4i | 0.85 (0.71-1.02) |  | 0.95(0.79-1.14) |  | 1.0 |  | 1.07 (0.87-1.16) |  |
| Placebo | *0.84 (0.76-0.94)* |  | 0.94 (0.84-1.06) |  | 0.99 (0.86-1.15) |  | 1.0 |  |
| **CV DEATH** |  |  |  |  |  |  |  |  |
| GLP-1RA | 1.0 |  | 0.99 (0.88-1.11) |  | 1.12 (0.98-1.32) |  | *1.15 (1.05-1.44)* |  |
| SGLT-2i | 1.01 (0.90-1.13) |  | 1.0 |  | 1.13 (1.0-1.30)  P value=0.047 |  | *1.16 (1.08-1.25)* |  |
| DPP-4i | 0.89 (0.76-1.02) |  | 0.88 (0.77-1.0)  P value=0.047 |  | 1.0 |  | 1.02 (0.98-1.14) |  |
| Placebo | *0.87 (0.80-0.95)* |  | *0.86 (0.80-0.92)* |  | 0.98 (0.88-1.08) |  | 1.0 |  |
| **TOTAL DEATH** |  |  |  |  |  |  |  |  |
| GLP-1RA | 1.0 |  | 1.0 (0.90-1.10) |  | *1.15 (1.01-1.30)* |  | *1.14 (1.05-1.23)* |  |
| SGLT-2i | 1.0 (0.91-1.11) |  | 1.0 |  | *1.15 (1.02-1.29)* |  | *1.14 (1.07-1.21)* |  |
| DPP-4i | *0.87 (0.77-0.99)* |  | *0.87 (0.77-0.98)* |  | 1.0 |  | 0.99 (0.90-1.09) |  |
| Placebo | *0.88 (0.81-0.95)* |  | *0.88 (0.82-0.93)* |  | 1.01 (0.92-1.11) |  | 1.0 |  |
| **HFH** |  |  |  |  |  |  |  |  |
| GLP-1RA | 1.0 |  | *0.76 (0.68-0.85)* |  | *1.17 (1.03-1.34)* |  | *1.11 (1.03-1.20)* |  |
| SGLT-2i | *1.32 (1.18-1.46)* |  | 1.0 |  | *1.54 (1.37-1.74)* |  | *1.45 (1.37-1.56)* |  |
| DPP-4i | *0.85 (0.75-0.97)* |  | *0.65 (0.57-0.73)* |  | 1.0 |  | 0.94 (0.85-1.04) |  |
| Placebo | *0.90 (0.83-0.98)* |  | *0.69 (0.64-0.73)* |  | 1.06 (0.96-1.17) |  | 1.0 |  |
| **RENAL outcome** |  |  |  |  |  |  |  |  |
| GLP-1RA | 1.0 |  | *0.78 (0.68-0.91)* |  | *1.28 (1.03-1.59)* |  | *1.21 (1.08-1.35)* |  |
| SGLT-2i | *1.28 (1.10-1.50)* |  | 1.0 |  | *1.65 (1.33-2.04)* |  | *1.56 (1.39-1.72)* |  |
| DPP-4i | *0.78 (0.63-0.97)* |  | *0.60 (0.49-0.74)* |  | 1.0 |  | 0.94 (0.78-1.13) |  |
| Placebo | *0.82 (0.74-0.92)* |  | *0.64 (0.58-0.72)* |  | 1.06 (0.88-1.27) |  | 1.0 |  |

The values in *Italics* indicate significant differences for the Risk Ratios; the values highlighted in yellow describe the P-value

calculated for the RR whose superior or inferior 95% CI intercept the Unit.

**PRISMA checklist**

| **Section/topic** | | | **#** | **Checklist item** | **Reported on page #** |  |
| --- | --- | --- | --- | --- | --- | --- |
| **TITLE** | | | | |  |  |
| Title | | | 1 | Identify the report as a systematic review, meta-analysis, or both. | 1 |  |
| **ABSTRACT** | | | | |  |  |
| Structured summary | | | 2 | Provide a structured summary including, as applicable: background; objectives; data sources; study eligibility criteria, participants, and interventions; study appraisal and synthesis methods; results; limitations; conclusions and implications of key findings; systematic review registration number. | 3 |  |
| **INTRODUCTION** | | | | |  |  |
| Rationale | | | 3 | Describe the rationale for the review in the context of what is already known. | 5-6 |  |
| Objectives | | | 4 | Provide an explicit statement of questions being addressed with reference to participants, interventions, comparisons, outcomes, and study design (PICOS). | 5-6 |  |
| **METHODS** | | | | |  |  |
| Protocol and registration | 5 | | | Indicate if a review protocol exists, if and where it can be accessed (e.g., Web address), and, if available, provide registration information including registration number. | 6 |  |
| Eligibility criteria | 6 | | | Specify study characteristics (e.g., PICOS, length of follow-up) and report characteristics (e.g., years considered, language, publication status) used as criteria for eligibility, giving rationale. | 6,7 |  |
| Information sources | 7 | | | Describe all information sources (e.g., databases with dates of coverage, contact with study authors to identify additional studies) in the search and date last searched. | 6,7 |  |
| Search | 8 | | | Present full electronic search strategy for at least one database, including any limits used, such that it could be repeated. | 6 |  |
| Study selection | 9 | | | State the process for selecting studies (i.e., screening, eligibility, included in systematic review, and, if applicable, included in the meta-analysis). | 6,7 |  |
| Data collection process | 10 | | | Describe method of data extraction from reports (e.g., piloted forms, independently, in duplicate) and any processes for obtaining and confirming data from investigators. | 6,7 |  |
| Data items | 11 | | | List and define all variables for which data were sought (e.g., PICOS, funding sources) and any assumptions and simplifications made. | 6,7 |  |
| Risk of bias in individual studies | 12 | | | Describe methods used for assessing risk of bias of individual studies (including specification of whether this was done at the study or outcome level), and how this information is to be used in any data synthesis. | 6,7 |  |
| Summary measures | 13 | | | State the principal summary measures (e.g., risk ratio, difference in means). | 7 |  |
| Synthesis of results | 14 | | | Describe the methods of handling data and combining results of studies, if done, including measures of consistency (e.g., I^2^) for each meta-analysis. | 7 |  |
| Risk of bias across studies | | 15 | | | Specify any assessment of risk of bias that may affect the cumulative evidence (e.g., publication bias, selective reporting within studies). | 7 |
| Additional analyses | | 16 | | | Describe methods of additional analyses (e.g., sensitivity or subgroup analyses, meta-regression), if done, indicating which were pre-specified. | 8 |
| **RESULTS** | | | | | |  |
| Study selection | | 17 | | | Give numbers of studies screened, assessed for eligibility, and included in the review, with reasons for exclusions at each stage, ideally with a flow diagram. | 9, Figure S1 |
| Study characteristics | | 18 | | | For each study, present characteristics for which data were extracted (e.g., study size, PICOS, follow-up period) and provide the citations. | 9; (Table 1) |
| Risk of bias within studies | | 19 | | | Present data on risk of bias of each study and, if available, any outcome level assessment (see item 12). | (Figure S2, Table S1) |
| Results of individual studies | | 20 | | | For all outcomes considered (benefits or harms), present, for each study: (a) simple summary data for each intervention group (b) effect estimates and confidence intervals, ideally with a forest plot. | 9,10 |
| Synthesis of results | | 21 | | | Present results of each meta-analysis done, including confidence intervals and measures of consistency. | 9,10 |
| Risk of bias across studies | | 22 | | | Present results of any assessment of risk of bias across studies (see Item 15). | 9-11 |
| Additional analysis | | 23 | | | Give results of additional analyses, if done (e.g., sensitivity or subgroup analyses, meta-regression [see Item 16]). | 9-11 |
| **DISCUSSION** | | | | | |  |
| Summary of evidence | | | 24 | | Summarize the main findings including the strength of evidence for each main outcome; consider their relevance to key groups (e.g., healthcare providers, users, and policy makers). | 12 |
| Limitations | | | 25 | | Discuss limitations at study and outcomelevel (e.g., risk of bias), and at review-level (e.g., incomplete retrieval of identified research, reporting bias). | 12-14 |
| Conclusions | | | 26 | | Provide a general interpretation of the results in the context of other evidence, and implications for future research. | 12-15 |
| **FUNDING** | | | | | |  |
| Funding | | | 27 | | Describe sources of funding for the systematic review and other support (e.g., supply of data); role of funders for the systematic review. | 15 |
